# Comparison of cord blood hematological parameters among normal, α-thalassemia, and β-thalassemia fetuses between 17 and 38 weeks of gestation

**DOI:** 10.1038/s41598-021-82297-y

**Published:** 2021-02-15

**Authors:** Wenli Zhan, Hao Guo, Siqi Hu, Jicheng Wang, Danqing Qin, Juqing Liang, Li Du, Mingyong Luo

**Affiliations:** 1 Medical Genetic Center, Guangdong Women and Children’s Hospital, Guangzhou Medical University, 521-523 Xingnan Avenue, Panyu District, Guangzhou, 511442 People’s Republic of China; 2 Medical Genetic Center, Guangdong Women and Children Hospital, Guangzhou, People’s Republic of China

**Keywords:** Genetic testing, Molecular medicine, Haematological diseases

## Abstract

The aim of this study was to retrospectively compare hematological parameters among normal, α-, and β-thalassemia fetuses between 17 and 38 weeks of gestation. Pregnant women at risk of having fetuses with thalassemia major and underwent cordocentesis for prenatal diagnosis were recruited. Fetal cord blood samples were collected from 249 fetuses for hematological and DNA analysis. Fetuses were divided into subgroups according to thalassemia DNA genotypes. The average and gestational age of subjects were 27.95 ± 5.78 years and 27.78 ± 3.57 weeks, respectively. The distribution of α-thalassemia, β-thalassemia, and normal cases was 67.87%, 19.68%, and 12.45%, respectively. Significant differences in almost all the hematological parameters (HbF, HbA, Hb, HCT, MCV, MCH, MCHC, RDW, and NBRCs) were observed in three groups (*P* < 0.001, except for RBC, *P* = 0.446). These differences were also observed in four α-thalassemia subgroups (*P* < 0.001) and were associated with the number of defected genes. Similarly, in five β-thalassemia genotypes, HbF, HbA, RBC, MCV, MCH and NBRCs were presented differently (*P* < 0.05). Additionally, the trends in RBC, Hb, and HCT changes in three α-thalassemia subgroups (silent carrier, trait, and major) and β^+^/β^+^ fetuses’ MCV, MCH, and RDW levels with gestation age were opposite to those of normal fetuses. We compared the distribution of hematological parameters in fetuses affected by most genotypes of thalassemia, as well as their trends in relation to gestational age for the first time, which is a good reference for future studies and prenatal diagnostic practices. The investigated hematological parameters are also valuable in diagnosing and differentiating thalassemia.

## Introduction

Thalassemia is a medical condition caused by mutations or deletions of globin genes, which results in abnormal hemoglobin (Hb) formation, causing red blood cell embrittlement and hemolytic anemia disease^1^. According to the specific defects of the relevant globin genes, thalassemia is classified into two major types, namely α- and β-thalassemia. The severity of the disease is dependent on the degree of imbalance in the quantity of the globin chains. Fetuses with thalassemia major, the most severe form of thalassemia, will either die or be born with serious complications, which heavily threaten the health of both the fetuses and the mothers. Thalassemia mainly occurs in tropical and sub-tropical areas such as Mediterranean countries, the Indian subcontinent, the Middle East, North Africa, and Southeast Asia^2,3^. In China, the overall prevalence of α-thalassemia and β-thalassemia was reported as 7.88% and 2.21%, respectively^4–6^. Therefore, it is a considerable burden to families of thalassemia major patients and their societies. To relief from such burden, effective measures to prevent and control prevalence of thalassemia are absolutely vital.

Nowadays, several programs, including carrier screening, molecular diagnostics, and prenatal diagnosis have been applied to screen α^0^-thalassemia (the most severe form of α-thalassemia) carriers or β-thalassemia minor to prevent the development of thalassemia major in the prevalent regions^2^. Among these, prenatal diagnosis is essential because the detection of fetal cases provides direct evidence for whether and how the fetuses are affected. Currently, extensive knowledge and experience have been accumulated in the prenatal diagnosis of thalassemia, but little is known about the distribution of red blood cell indices in the thalassemia fetuses. Although previous studies have shed light on the hematological values in normal fetuses between 18 and 30 weeks of gestation, fetuses with α- and β-thalassemia and their subgroups related to severity, were not included in the analysis^7,8^. Moreover, in a study where red blood cell indices such as Hb, mean corpuscular volume (MCV), mean corpuscular hemoglobin (MCH), and mean cell hemoglobin concentration (MCHC) were investigated among normal, α-thalassemia-1 trait^9^ (two alpha gene defects), and Hb Barts fetuses, it revealed that these basic data are valuable in differentiating the affected fetuses among couples at risk^10^. Therefore, it is worthy to explore the role of hematological indices (not limited to Hb, MCV, MCH, and MCHC) in α- and β-thalassemia and their different genotypes.

The aim of this study was to cast light on the differences in hematological values of fetuses with α- and β-thalassemia and their subgroups with varying severity between 17 and 38 weeks of gestation. Besides, the effect of gestational weeks on these hematological values was also observed.

## Results

### Characteristics of the subjects

A total of 247 pregnant women with 248 fetuses were recruited in this study. The average age and gestational age were 27.95 ± 5.78 years and 27.78 ± 3.57 weeks, respectively (data not shown). The ratio of α-thalassemia, β-thalassemia, and normal cases was 67.87% (169/249), 19.68% (49/249), and 12.45% (31/249), respectively. The detailed distribution of different thalassemia genotypes is shown in Table 1. No significant differences were observed in age (*P* = 0.069) and gestational age (*P* = 0.192) among the three groups (data not shown).

**Table 1:** The distribution of genotype in α-thalassemia and β-thalassemia.

	Genotype	N (case)	Total N (case)
**α-thalassemia**			
α-thalassemia silent carrier (one alpha-gene defect)			
(-α/αα)	(-α^3.7^/αα)	6	15
	(-α^4.2^/αα)	4	
(α^T^α/αα)	(α^WS^ α/αα)	2	
	(α^CS^α/αα)	3	
α-thalassemia trait (two alpha-gene defects)			
(--/αα)	(--^SEA^/αα)	28	31
	(-α^3.7^/-α^3.7^)	1	
(α^T^α/α^T^α)	(α^CS^α/α^CS^α)	2	
α-thalassemia intermedia (three alpha-gene defects)			
(--/-α)	(-α^3.7^/ )	7	21
	(-α^4.2^/ )	6	
	(-α^3.7^/--^THAI^)	1	
(--/α^T^α)	(α^CS^α/)	4	
	(α^QS^α/)	2	
	(α^WS^α/)	1	
α-thalassemia major (four alpha-gene defects)			
(--/--)	(--^SEA^/)	102	102
**β-thalassemia**			
β^+^/β^N^	β^28^/β^N^	2	8
	β^IVS-II-654^/β^N^	6	
β^0^/β^N^	β^CD14–15^/β^N^	1	16
	β^CD27–28^/β^N^	2	
	β^CD41–42^/β^N^	11	
	β^CD71–72^/β^N^	2	
β^+^/β^+^	β^28^/β^28^	1	3
	β^28^/β^IVS-II-654^	1	
	β^28^/β^IVS-II-672^	1	
β^0^/β^+^	β^CD41–42^/β^28^	6	15
	β^CD17^/β^28^	1	
	β^CD17^/β^IVS-II-654^	2	
	β^CD41–42^/β^IVS-II-654^	5	
	β^CD27–28^/β^IVS-II-654^	1	
β^0^/β^0^	β^CD17^/β^CD41–42^	3	7
	β^CD41–42^/β^CD41–42^	2	
	β^CD71–72^/β^CD17^	2	

### Variety and distribution of hemoglobin fractions in fetuses of different genotypes

Fetuses with α-thalassemia presented several Hb fractions including HbF, HbA, HbA_2_, Hb Barts, Hb CS, Hb Portland, Hb Epsilon4, Hb Gower1, HbH, and other HbF variant (Table 2). HbF, HbA, and Hb Barts were present in every α-thalassemia fetus, while other Hb fractions were only present in some of the α-thalassemia fetuses. In β-thalassemia fetuses, only HbF and HbA were present (Table 3). As shown in Table 3, significant differences were observed in HbF and HbA levels (*P* < 0.001, *P* < 0.001, respectively) among different types of thalassemia and normal fetuses.

**Table 2:** Distribution of hemoglobin fractions presented in 169 α-thalassemia fetuses (%).

	Genotype	N (cases)	HbF	HbA	HbA_2_	Hb Barts	Hb CS	HbF variant	Hb Portland	Hb Epsilon4	Hb Gower1	HbH
α-thalassemia	α-thalassemia silent carrier (one alpha-gene defect)	15	91.49 ± 3.01	7.74 ± 2.69	0.1 (1 case)	0.81 ± 0.66	–	–	–	–	–	–
	α-thalassemia trait (two alpha-gene defects)	31	83.75 ± 9.88	9.46 ± 3.71	0.3 ± 0.08 (4 cases)	4.71 ± 2.81	1.10–2.50 (2 cases)	5.70–54.90 (2 cases)	1.30 (1 case)	–	–	–
	α-thalassemia intermedia (three alpha-gene defects)	21	61.76 ± 10.13	11.62 ± 3.46	0.20–0.40 (2 cases)	23.52 ± 5.63	1.50 ± 0.56 (4 cases)	46.20 (1 case)	0.74 ± 0.17 (10 cases)	0.52 ± 0.17 (9 cases)	–	0.20
	α-thalassemia major (four alpha-gene defects)	102	–	–	–	86.02 ± 3.79	–	12.33 ± 1.72 (3 cases)	11.67 ± 3.0	1.55 ± 0.21 (39 cases)	4.20 ± 2.15 (39 cases)	0.91 ± 0.44 (26 cases)

**Table 3:** Distribution and Comparisons of hematological parameters among fetuses of different genotypes.

Items	α-thalassemia						β-thalassemia							Normal group (31 cases)	*P *^*c*^ value
	α-thalassemia silent carrier (15 cases)	α-thalassemia trait (31 cases)	α-thalassemia intermedia (21 cases)	α-thalassemia major (69 cases)	Tota1 α-thalassemia (136 cases)	*P *^*a*^ value	β^0^/β^N^ (16 cases)	β^+^/β^N^ (8 cases)	β^+^/β^+^ (3 cases)	β^0^/β^0^ (7 cases)	β^0^/β^+^ (15 cases)	Total β-thalassemia (49 cases)	*P *^*b*^ value		
Age (years)	27.40 ± 4.88	27.25 ± 5.69	26.71 ± 6.02	27.65 ± 5.37	27.39 ± 5.45	0.961	26.75 ± 7.64	27.00 ± 4.81	29.00 ± 3.74	28.57 ± 6.68	28.60 ± 5.34	27.87 ± 5.93	0.610	30.10 ± 6.51	**0.050**
Gestational age (weeks)	29.40 ± 2.41	28.22 ± 2.28	28.90 ± 3.52	28.67 ± 2.25	28.68 ± 2.50	0.484	30.38 ± 3.85	29.00 ± 2.27	32.00 ± 5.89	23.86 ± 1.77	24.47 ± 3.36	27.76 ± 4.45	** < 0.001**	28.77 ± 3.46	0.103
HbF (%)	91.49 ± 3.01	83.75 ± 9.88	61.76 ± 10.13	–	78.67 ± 14.71	** < 0.001**	96.40 ± 1.66	96.76 ± 0.74	97.05 ± 4.89	100 ± 0	99.69 ± 0.83	98.00 ± 2.28	** < 0.001**	93.08 ± 2.54	** < 0.001**
HbA (%)	7.74 ± 2.69	9.46 ± 3.71	11.61 ± 3.46	–	9.75 ± 3.67	**0.005**	3.58 ± 1.67	3.24 ± 0.74	5.90 ± 6.08	–	1.15 ± 1.38	3.32 ± 2.05	** < 0.001**	6.92 ± 2.54	** < 0.001**
Hb Barts (%)	0.81 ± 0.66	4.71 ± 2.81	23.52 ± 5.63	86.70 ± 3.48	47.96 ± 39.34	** < 0.001**	–	–	–	–	–	–	–	–	–
RBC (10*^12^/L)	3.99 ± 1.11	3.89 ± 0.78	3.65 ± 0.84	3.02 ± 0.57	3.43 ± 0.84	** < 0.001**	3.90 ± 0.45	3.72 ± 0.45	3.68 ± 0.30	3.25 ± 0.29	3.23 ± 0.34	3.58 ± 0.47	**0.003**	3.59 ± 0.53	0.446
Hb (g/L)	127.53 ± 11.56	117.31 ± 22.61	91.14 ± 17.35	69.96 ± 11.39	90.87 ± 27.88	** < 0.001**	139.50 ± 16.80	139.25 ± 11.16	135.33 ± 20.21	127.71 ± 8.07	125.86 ± 10.17	133.02 ± 13.33	0.086	129.42 ± 15.94	** < 0.001**
HCT (%)	42.02 ± 10.57	38.42 ± 7.13	32.68 ± 5.29	29.65 ± 4.85	33.58 ± 7.79	** < 0.001**	42.79 ± 4.15	41.93 ± 3.39	40.50 ± 6.20	39.51 ± 2.36	38.29 ± 2.86	40.69 ± 3.71	0.074	39.79 ± 4.72	** < 0.001**
MCV (fL)	105.78 ± 4.46	99.89 ± 10.06	91.59 ± 11.09	98.69 ± 7.76	98.66 ± 9.35	** < 0.001**	110.24 ± 7.16	113.11 ± 6.86	109.57 ± 7.87	122.11 ± 7.87	119.20 ± 7.55	114.43 ± 8.86	**0.020**	111.93 ± 11.28	** < 0.001**
MCH (pg)	33.03 ± 4.39	30.34 ± 1.98	25.36 ± 2.87	23.21 ± 1.74	26.33 ± 4.42	** < 0.001**	35.91 ± 3.10	37.55 ± 2.09	36.63 ± 2.48	39.47 ± 2.33	39.15 ± 2.28	37.40 ± 3.00	**0.031**	36.51 ± 3.79	** < 0.001**
MCHC (g/L)	296.80 ± 64.85	304.69 ± 13.60	277.95 ± 18.02	235.37 ± 11.53	265.25 ± 39.38	** < 0.001**	325.79 ± 18.23	332.00 ± 3.25	334.67 ± 1.53	323.29 ± 7.39	328.79 ± 12.00	327.00 ± 13.00	0.560	326.10 ± 10.74	** < 0.001**
RDW (%)	15.54 ± 1.26	16.81 ± 3.18	19.77 ± 3.64	26.76 ± 5.25	22.07 ± 6.42	** < 0.001**	16.31 ± 1.21	16.50 ± 0.69	16.63 ± 1.27	16.04 ± 0.63	16.01 ± 1.20	16.13 ± 1.04	0.451	15.78 ± 1.15	** < 0.001**
NRBCs (%)	13.65 (8.48–20.13)	11.3 (5.00–24.90)	29.6 (13.93–77.10)	1540.7 (1055.75–2049.00	212.80 (14.00–1555.00)	** < 0.001**	9.90 (6.60–23.20)	13.0 (6.30–18.0)	5.05 (2.80–7.30)	22.35 (11.2–37.85)	11.0 (9.10–23.30)	12.70 (7.30–25.40)	**0.012**	2.5 (0.1405–5.875)	** < 0.001**

*P*^*a*^: Comparison among 4 α-thalassemia subgroups using ANOVA test for all the hematological parameters except for the NRBCs using Kruskal–Wallis test; *P*^*b*^: comparison among 5 β-thalassemia subgroups using ANOVA test for all the hematological parameters except for the NRBCs using Kruskal–Wallis test; *P*^*c*^: comparison among all the α-thalassemia cases, all the β-thalassemia cases and normal group using ANOVA test for all the hematological parameters except for the NRBCs using Kruskal–Wallis test. Significant differences (*P* < 0.05) are indicated in bold.

### Comparison of hematological parameters between fetuses of different genotypes

**Figure 1:**
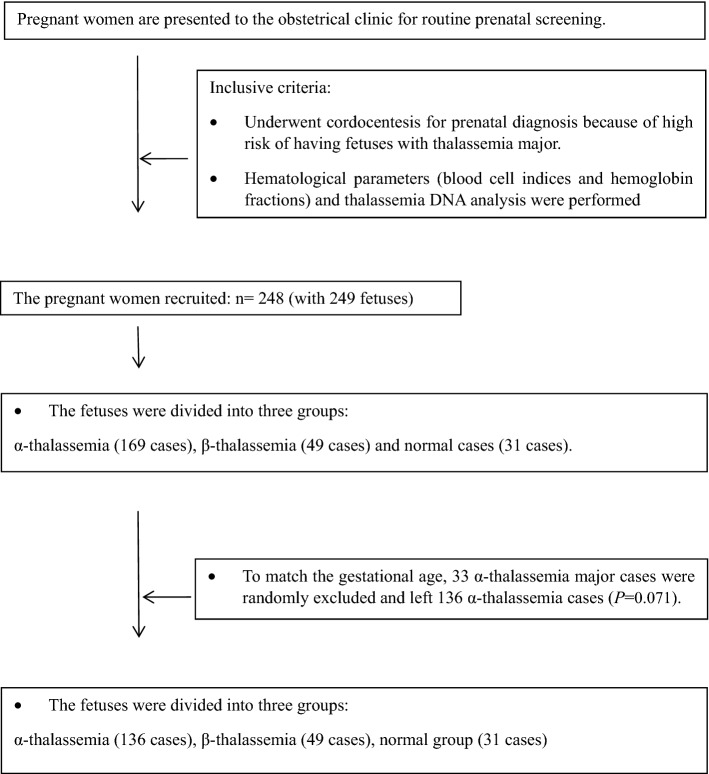
Flow chart of the experimental process for the subjects recruited in this study.

In the subgroups of α-thalassemia and β-thalassemia, significant differences were observed in gestational age (*P* = 0.003 and *P* < 0.001, respectively, data not shown). It is well known that the hematological parameters of a fetus change with different gestational ages. To control for this factor, the α-thalassemia major (four alpha-gene defects) group was selected randomly to match the gestational age with other groups (*P* = 0.071, as mentioned in Fig. 1). The β-thalassemia subgroups were not adjusted for the limited sample sizes.

Significant differences were observed in hematological parameters including Hb, HCT, MCV, MCH, MCHC, RDW, and percentage of NRBCs among the three groups (*P* < 0.001 for all the groups, Table 3). Furthermore, in the five subgroups of β-thalassemia, significant differences were found in RBC, MCV, MCH levels and percentage of NRBCs (*P* = 0.003, *P* = 0.020, *P* = 0.031, and *P* = 0.012, respectively).

As for the four subgroups of α-thalassemia, significant differences were found in all the hematological parameters listed (*P* ≤ 0.005, Table 3). To explore the association between deletion α-thalassemia and mutant α-thalassemia, the four α-thalassemia groups were further divided into seven subgroups. As illustrated in Table 4, no significant differences were found between the α-thalassemia silent carrier (one alpha-gene defect) subgroups, but in the subgroups of α-thalassemia trait (two alpha-gene defects) and α-thalassemia intermedia (three alpha-gene defects), significant differences were found in Hb Barts, RBC, Hb, HCT, MCV, and percentage of NRBCs. In terms of the levels of Hb Barts, MCV, and the proportion of NRBCs, in contrast to the levels of RBC, Hb, and HCT, the levels in mutant groups were higher than those of deletion groups. Furthermore, a significant difference was found in MCH levels between α-thalassemia intermedia subgroups, and also in MCHC and RDW levels between α-thalassemia trait subgroups.

**Table 4:** Comparison of hematological parameters between fetuses of 4 different α-thalassemia genotypes.

Items	α-thalassemia silent carrier (one alpha-gene defect)			α-thalassemia trait (two alpha-gene defects)			α-thalassemia intermedia (three alpha-gene defects)			α-thalassemia major (four alpha-gene defects) (69 cases)	*P*^*d*^ value
	(-α/αα) (10 cases)	(α^T^α/αα) (5 cases)	*P*^*a*^ value	(--/αα) (28 cases)	(α^T^α/α^T^α) (3 cases)	*P*^*b*^ value	(--/-α) (14 cases)	(--/α^T^α) (7 cases)	*P*^*c*^ value		
Age (years)	28.10 ± 5.11	26.00 ± 4.58	0.453	27.04 ± 5.70	29.00 ± 7.55	0.584	26.93 ± 6.82	26.29 ± 4.42	0.824	27.65 ± 5.37	0.961
Gestational age (weeks)	29.20 ± 2.25	29.80 ± 2.95	0.667	28.11 ± 2.38	28.67 ± 1.53	0.695	29.64 ± 2.82	27.43 ± 4.50	0.181	28.67 ± 2.25	0.484
HbF (%)	91.38 ± 2.59	91.72 ± 4.07	0.846	83.37 ± 5.62	84.37 ± 5.62	0.872	61.84 ± 10.40	61.61 ± 10.39	0.964	–	** < 0.001**
HbA (%)	8.00 ± 2.40	7.22 ± 3.44	0.614	9.95 ± 3.66	6.57 ± 1.79	0.128	12.34 ± 3.56	10.17 ± 2.98	0.182	–	**0.005**
Hb Barts (%)	0.62 ± 0.53	1.33 ± 0.78	0.081	4.43 ± 2.31	7.87 ± 5.72	**0.044**	21.80 ± 2.28	26.97 ± 8.58	**0.044**	86.70 ± 3.48	** < 0.001**
RBC (10*^12^/L)	4.27 ± 1.27	3.45 ± 0.26	0.185	3.97 ± 0.64	2.96 ± 1.55	**0.032**	4.11 ± 0.50	2.71 ± 0.49	** < 0.001**	3.02 ± 0.57	** < 0.001**
Hb (g/L)	131.40 ± 11.15	119.80 ± 8.70	0.064	119.82 ± 19.10	87.67 ± 37.69	**0.017**	99.86 ± 11.00	73.71 ± 14.48	** < 0.001**	69.96 ± 11.39	** < 0.001**
HCT (%)	44.44 ± 12.28	37.18 ± 2.80	0.222	39.15 ± 6.38	30.13 ± 10.83	**0.037**	35.46 ± 3.81	27.11 ± 2.72	** < 0.001**	29.65 ± 4.85	** < 0.001**
MCV (fL)	104.72 ± 4.61	107.90 ± 3.66	0.204	98.77 ± 4.95	112.20 ± 31.27	**0.027**	86.41 ± 3.73	101.93 ± 13.87	**0.001**	98.69 ± 7.76	** < 0.001**
MCH (pg)	32.14 ± 5.21	34.80 ± 0.73	0.285	30.25 ± 1.51	31.37 ± 5.31	0.369	24.30 ± 0.88	27.49 ± 4.24	**0.012**	23.21 ± 1.74	** < 0.001**
MCHC (g/L)	283.98 ± 77.43	322.40 ± 4.51	0.152	306.46 ± 10.20	284.67 ± 27.65	**0.006**	281.93 ± 0.90	270.00 ± 26.71	0.158	235.37 ± 11.53	** < 0.001**
RDW (%)	15.27 ± 1.29	16.08 ± 1.11	0.253	16.32 ± 1.65	21.83 ± 8.81	**0.003**	18.97 ± 2.11	21.37 ± 5.48	0.159	26.76 ± 5.25	** < 0.001**
NRBCs (%)	13.10 (9.90–17.90)	18.40 (7.40–21.0)	0.303	11.0 (5.03–23.30)	212.80 (3.3–6312.3)	**0.001**	16.45 (12.58–25.75)	661.50 (309.60–2548.25)	**0.025**	1540.7 (1055.75–2049.00	** < 0.001**

*P*^*a*^, *P*^*b*^, *P*^*c*^ were the *P* values of comparison between (-α/αα) and (α^T^α/αα) group, (--/αα) and (α^T^α/α^T^α) group, (--/-α) and (--/α^T^α) group using Student's t test for all the hematological parameters except for the NRBCs using Mann–Whitney test, respectively. *P*^*d*^ were the *P* values of comparision among all the α subgroups using Student's t test for all the hematological parameters except for the NRBCs using Mann–Whitney test. Significant differences (*P* < 0.05) are indicated in bold.

### Distribution of hematological parameters in fetuses of different genotypes at different gestational ages

**Figure 2:**
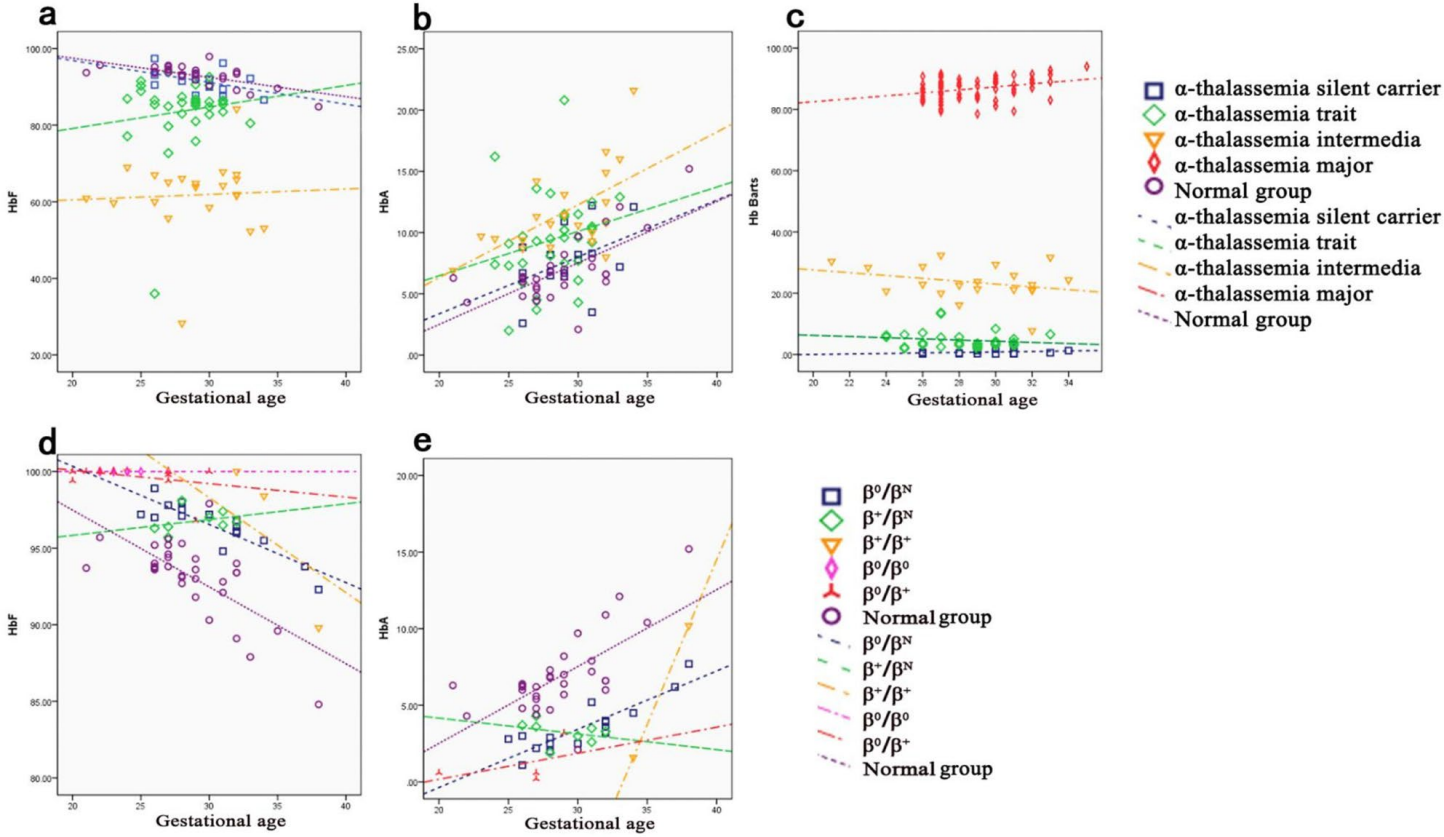
Distribution of hemoglobin fractions in four α-thalassemia genotypes and five β-thalassemia genotypes at different gestational ages. The trends of HbF (**a**), HbA (**b**), Hb Barts (**c**) with increased gestational ages in four α-thalassemia genotypes carriers and normal group were illustrated, as well as trends of HbF (**d**), HbA (**e**) in five β-thalassemia genotypes carriers and normal group.

The hematological parameters varied with gestational age and number of defected gene. First, the level of Hb fractions varied with gestation age and number of defected thalassemia gene in most instances (Fig. 2). The HbF concentration declined with increased gestation age in all groups, except for the α-thalassemia trait and intermedia groups. Similarly, Hb Barts concentration declined with increased gestation age except for the α-thalassemia major group. However, the level of HbA increased with gestation age in all groups, except in the β^+^/β^N^ groups. Moreover, with an increase in the number of α-thalassemia defected genes, HbF levels decreased, but HbA and Hb Barts increased. Contrarily, in the β-thalassemia groups, increased defected gene number lead to increased HbF but decreased HbA levels.

**Figure 3:**
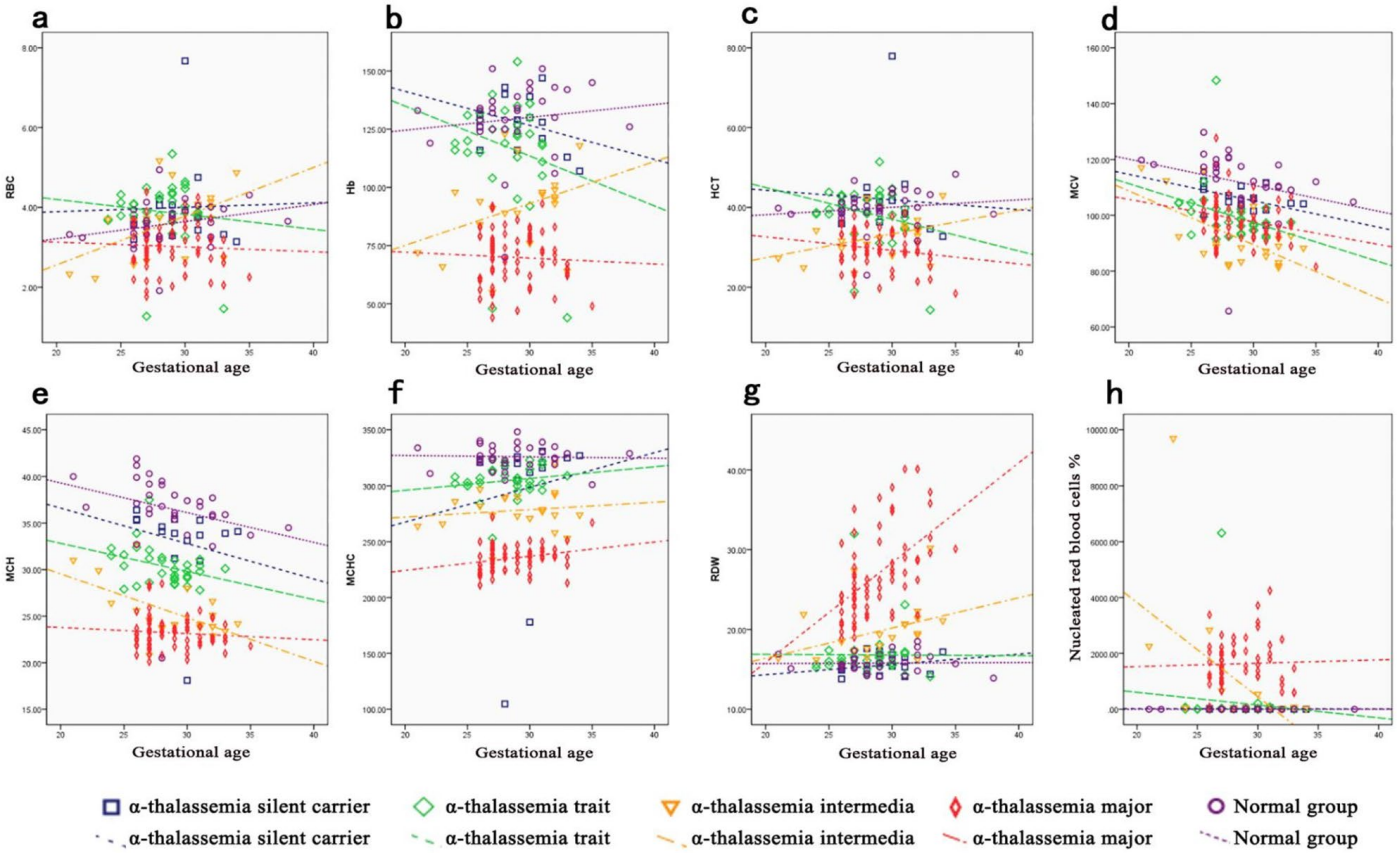
Distribution of red blood cell indices in four α-thalassemia genotypes at different gestational ages. The trends of RBC (**a**), Hb (**b**), HCT (**c**), MCV (**d**), MCH (**e**), MCHC (**f**), RDW (**g**) and nucleated erythrocytes (**h**) with increased gestational ages in four α-thalassemia genotypes carriers and normal group were illustrated.

**Figure 4:**
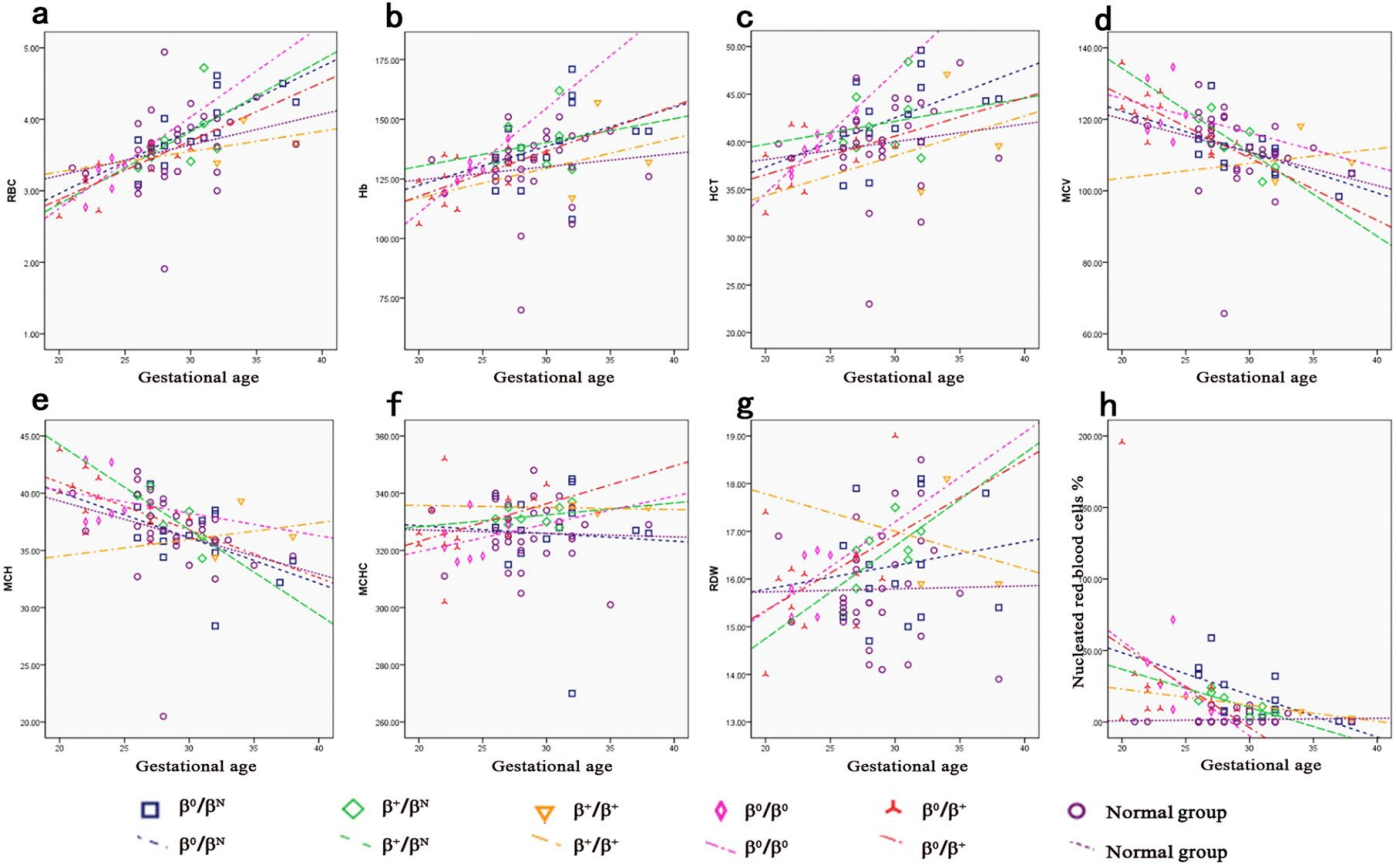
Distribution of red blood cell indices in five β-thalassemia genotypes at different gestational ages. The trends of RBC (**a**), Hb (**b**), HCT (**c**), MCV (**d**), MCH (**e**), MCHC (**f**), RDW (**g**) and nucleated erythrocytes (**h**) with increased gestational ages in five β-thalassemia genotypes carriers and normal group were illustrated.

Second, the red blood cell indices changed with gestation age and number of α- and β-thalassemia defected gene as well (Figs. 3 and 4, respectively). Specifically, in the normal groups, with the increase in gestation age, there were upward trends in RBC, Hb, HCT, while the levels of MCV, MCH, and MCHC decreased, RDW and NRBCs kept constantly. In all the α-thalassemia subgroups, tendencies of MCV and MCH decreased with GA as those of normal fetuses, while trend of MCHC were opposite (Fig. 3). The trends of RBC, Hb and HCT in the α-thalassemia intermedia group were the same as those of the normal groups, while in other α-thalassemia groups were opposite (Fig. 3). In β-thalassemia affected groups, the trends in change of all the red blood cell indices were the same as those of normal groups except for MCV, MCH, and RDW levels in the β^+^/β^+^ groups (Fig. 4).

As for the associations between red blood cell indices and the severity of defected thalassemia gene, it was obvious that groups with more α-thalassemia genes defected had lower levels of RBC, Hb, HCT, MCV, MCH, and MCHC but higher levels of RDW and NRBCs. However, among groups with different number of defected β-thalassemia genes, such associations between red blood cell indices and the severity of β-thalassemia were not significant, though significant differences were observed in the level of HbF, HbA, RBC, MCV, MCH, and NRBCs (Table 3).

## Discussion

Hematological indices have previously been suggested to have a potential role in differentiating thalassemia in affected fetuses^10^. However, the previous study is limited by the fact that only finite hematological indices were studied in fetuses affected by α-thalassemia only, and not β-thalassemia and its different genotype subgroups. Therefore, the purpose of this study is to investigate the levels of hematological indices in fetuses with α- and β-thalassemia and their subgroups with varying genotypes between 17 and 38 weeks of gestation, along with how these hematological values range with differences in gestation age.

In this study, the distribution of Hb Barts disease was 40.96% (102/249). The incidence of Hb Barts disease was relatively higher than that in the study performed by Kasemsri et al*.* (29.5%) in Thailand^10^. This difference may be due to the different gestational age ranges of the recruited subjects between their study (18–22 weeks) and our study (17–38 weeks). Some Hb Barts disease cases may have been overlooked due to the narrow gestational age range. Moreover, differences in the prevalence of thalassemia in different populations and sample sizes could also contribute to increase in incidence^11–13^. In our study, as for the genotype, 14 different α-thalassemia genotypes and 17 different β-thalassemia genotypes were found. Interestingly, a rare β^28^/β^IVS-II-672^ genotype was also found. Consistent with Ketong et al*.*’s study^6^, the most common α- and β-globin gene mutations were --^SEA^ and CD41/42, respectively.

As for the hemoglobin fractions, because β-thalassemia fetuses were rarely researched, our study showed for the first time that significant a difference was observed in the percentage of HbF in five different β-thalassemia genotypes (*P* < 0.001, Table 3). Moreover, HbF levels increased with the number of defected β-thalassemia genes, while the proportion of HbA decreased. Interestingly, HbF in β-thalassemia groups were significantly higher than those of normal groups, while HbA levels were lower than those of normal groups. These results indicate that HbF and HbA potentially play a role in differentiating the number of defected gene and indicated severity of β-thalassemia. In fetuses with α-thalassemia, Hb Barts was detected in every fetus, even though the levels were not high in silent carriers. All of the 102 fetuses with α-thalassemia major presented with Hb Barts (86.02 ± 3.79) and Hb Portland (11.67 ± 3.0), and 39 cases presented with Hb Epsilon4 (1.55 ± 0.21) and Hb Gower1 (4.20 ± 2.15) simultaneously. Additionally, HbH was detected in 26 fetuses with α-thalassemia major. Hb CS was found in 6 fetuses with CS mutations. As we all known, the defect of α-thalassemia gene leads to the reduction of normal alpha-globin chain and normal fetal hemoglobins. So in fetuses of alpha-thalassemia, these abnormal hemoglobins were produced for compensation because of the reduction of normal alpha-globin chain. Similar to previous studies,^1,14^ the proportion of HbF decreased with the severity of α-thalassemia, while the level of HbA, Hb Barts, Hb Portland, and Hb Epsilon4 increased. Therefore, since the percentage of HbF, HbA, and Hb Barts were significantly different among α-thalassemia, β-thalassemia, their different genotype subgroups, and normal fetuses in our study, and they also correlated with the number of affected genes, HbF, HbA, and Hb Barts is valuable in diagnosing and differentiating thalassemia.

When comparing the hematological parameters between fetuses of different genotypes, first, between fetuses with five different β-thalassemia genotypes, significant differences were observed in the percentage of RBC, MCV, MCH and percentage of NRBCs (*P* < 0.05). Among them, RBC were increased with the type of defected β-thalassemia gene, which indicates the potential role of these parameters in differentiating the severity of β-thalassemia. Similarly, in terms of RBC, Hb, HCT, MCV, MCH, MCHC, and RDW, significant differences were noticed between four different α-thalassemia subgroups (*P* < 0.001), and they all increased with the number of defected genes, except RDW which declined with the severity of defected genes. Hence, these indices could distinguish different α-thalassemia genotypes. Interestingly, our study showed for the first time that in α-thalassemia, although the same number of genes were affected, the hematological parameters presented differently between groups with mutation and deletion defects. As shown in Table 4, in the α-thalassemia trait and intermedia groups, the manifestation of fetuses with mutations was more severe than those with deletions, and they had higher Hb Barts levels, more severe anemia, lower RBC, HCT, and MCHC, and higher MCV and RDW. The differences between mutation and deletion defects may due to two reasons theoretically. One is that the compensatory function of the remaining normal α-gene in deleted α-thalassemia may be stronger than that of in mutated one. Another is that the mutated defects gene can produce abnormal hemoglobin which may directly damage red blood cells.Therefore, the hematological parameters could differentiate genotypes of thalassemia, and were valuable in the diagnosis of thalassemia. Since the relationships between hematological parameters of fetuses and thalassemia have rarely been studied, our study may serve as a reference for future studies and prenatal diagnosis practices.

Hematological parameters in the normal groups were observed to change with gestational age regularly and their changing trends were consistent with those in previous studies^7,8,15^. However, in fetuses with thalassemia, such associations between hematological parameters and gestation were rarely reported. In a previous study^16^, it was shown that the Hb level and the proportion of Hb Barts in fetuses of homozygous α^0^-thalassemia increased significantly with gestation. Their results of Hb levels were contradicting to our study, in which the Hb level in homozygous α^0^-thalassemia decreased with gestation. This contradiction may due to the sample size. For the relative study was rare, more studies and more samples were still needed. Moreover, we have shown for the first time that how the hematological indices range with differences in gestation age in four different α-thalassemia and five different β-thalassemia subgroups, and trends of RBC, Hb, and HCT in three α-thalassemia subgroups (silent carrier, trait, and major) were opposite to those of normal fetuses, while other subgroups were consistent to normal groups. In fetuses affected with β-thalassemia, the trends in changes of all the red blood cell indices were the same as those of normal fetuses, except for the MCV, MCH, and RDW levels of the β^+^/β^+^ subgroup. Our study supplied comprehensive information about the development of hematological parameters in different types and severities of thalassemia.

A limitation of this study is that the sample size is limited. As most thalassemia prevention and control programs, the pregnant women with high risk of having thalassemia major fetuses would be advised to undergo chorionic villus sampling or amniocentesis instead of cordocentesis in the first or second trimester. Therefore, the number of women having cordocentesis for prenatal diagnosis of thalassemia is limited. In addition, the subjects of this study were from Guangdong province only, subjects from more districts are needed in future studies.

To conclude, there are several strengths in this study. First, as far as we know, this is the first study that investigated the distribution of so many hematological parameters in fetuses affected by four α-thalassemia genotypes, five β-thalassemia genotypes, and normal fetuses, as well as the trends in changes with gestational age, which can be a good reference for future studies and prenatal diagnostic practices. Additionally, this study found that HbF, HbA, and Hb Barts have the value of diagnosing and differentiating thalassemia, as well as the RBC, MCV, MCH and percentage of NRBCs in β-thalassemia and RBC, Hb, HCT, MCV, MCH, MCHC, RDW in α-thalassemia. Lastly, we have shown for the first time that even with the same number of defected α-genes, the manifestation of fetuses with mutation defects was more severe than that of groups with deletion defects in fetuses. The hematologic studies among normal, α-, and β-thalassemia fetuses from this study supplied comprehensive information about the development of hematological parameters in different types and severities of thalassemia and may serve as a reference range for future studies or even clinical prenatal diagnosis applications.

## Materials and methods

### Subjects

All the pregnant women who were at risk of having fetuses with thalassemia major and underwent cordocentesis for prenatal diagnosis at the Guangdong Women and Children Hospital, Guangzhou, China, from 2014 to 2019 were recruited. This amounted to a total of 248 women with 249 fetuses. The experimental process for the subjects recruited in this study is shown in Fig. 1. Fetal cord blood samples were collected for analysis of hematological parameters (including blood cell indices and Hb fractions) and DNA analysis. The medical histories and informed consents were collected from all the subjects as well. The subjects were excluded when met with the following exclusion criteria: (1) cord blood samples could not be obtained for undergoing other prenatal diagnostic procedures instead of cordocentesis, or in case of the failure of cordocentesis; (2) the results of hematological parameters or thalassemia DNA analyzes were not complete; or (3) the medical histories and informed consents were not obtained.

All steps were performed according to the Declaration of Helsinki and informed consents were collected from all the participants. This research was approved by the Medical Ethics Committees of Guangdong Women and Children’s Hospital and Prenatal Diagnosis (Technology) Ethics Committee of Guangdong Women and Children’s Hospital. The prenatal diagnosis operations were performed under the ISUOG Practice Guidelines of invasive procedures for prenatal diagnosis^17^ (2016) and consistent with Implementation Rules for Prenatal Diagnosis Technology Management published by Guangdong Provincial government (http://wsjkw.gd.gov.cn/gkmlpt/content/2/2131/post_2131319.html#2531).

### Cordocentesis

Cordocentesis was performed by experienced perinatologists under the guidance of real-time ultrasound equipment (Aloka SSD-4000, Tokyo, Japan) with transabdominal 3.5 MHz curvilinear transducers. About 1.5 mL of fetal cord blood was collected from each subject for hematological and DNA analysis.

### Hematological parameter analysis

After mixing by inversion of the tubes, blood cell indices were measured from approximately 1 mL fetal cord blood with a Sysmex XN-1000 automated hematological analyzer (Sysmex, Tokyo, Japan), including red blood cell count (RBC), Hb levels, Hematocrit (HCT), MCV, MCH, MCHC, red cell distribution width (RDW), and the proportion of nucleated red blood cells (NRBCs).

### Hemoglobin fraction analysis

After dilution of 18 μL of fetal cord blood with 90 μL hemolytic agent (Sebia, Paris, France), the Hb fractions of the mixture was analyzed with a CAPILLARYS 2 automatic analyzer (Sebia).

### Genetic detection of thalassemia

All the fetal blood samples were subjected to a suspension array assay which was previously developed by our team^18^ and has been commercialized as the Thalassemia (α/β) Gene Diagnostic kit (DAAN Gene, Guangzhou, China). Mutations that were outside the detection range of the suspension array were detected by gene sequencing. All fetuses were divided into three groups: normal group; α-thalassemia; and β-thalassemia. Moreover, the latter two groups were further divided into four subgroups, namely the (-α/αα), (--/αα), (--/-α), and (--/--) genotype and five subgroups, the (β^+^/β^N^), (β^0/^β^N^), (β^+^/β^+^), (β^0^/β^+^), and (β^0^/β^0^) genotype, respectively.

### Statistical analyses

Normally distributed data are expressed as mean and standard deviation (SD), and the rest of the data are presented as median and interquartile range. Comparisons among three or more groups of the normally and informally distributed data were analyzed using the ANOVA test and Kruskal–Wallis test, respectively. Moreover, comparisons of mean and median differences between 2 groups were analyzed using Student's t test and Mann–Whitney test, respectively. Statistical analyses were carried out with SPSS software version 20.0. (IBM, New York, USA) and *P* < 0.05 was considered statistically significant.
